# Supplementary material for: High Phosphate-Induced JAK-STAT Signalling Sustains Vascular Smooth Muscle Cell Inflammation and Limits Calcification
Source: Biomolecules. 2023 Dec 24;14(1):29. doi: 10.3390/biom14010029 (PMC10813375; doi:10.3390/biom14010029)
Supplement: Supplementary file 1 [file biomolecules-14-00029-s001.zip › biomolecules-2494470-supplementary.pdf]

High phosphate-induced JAK-STAT signalling sustains Vascular Smooth Muscle Cells inflammation and limits calcification

Federica Macrì[1], Ilaria Vigorito[1], Stefania Castiglione[1], Stefano Faggiano[1], Manuel Casaburo[2], Nadia Fanotti[2], Luca Piacentini[3], Davide Vigetti[4], Maria Cristina Vinci[5] and Angela Raucci[1,2,#]

1 Unit of Experimental Cardio-Oncology and Cardiovascular Aging, Centro Cardiologico Monzino-IRCCS, 20138 Milan, Italy. 2 Animal Facility, Centro Cardiologico Monzino-IRCCS, 20138 Milan, Italy. 3 Bioinformatics and Artificial Intelligence Facility, Centro Cardiologico Monzino-IRCCS, 20138 Milan, Italy. 4 Department of Medicine and Surgery, University of Insubria, 21100 Varese, Italy. 5 Vascular Biology and Regenerative Medicine Unit, Centro Cardiologico Monzino-IRCCS, 20138 Milan, Italy.

# Corresponding author: Angela Raucci, Unit of Experimental Cardio-Oncology and Cardiovascular Aging, Centro Cardiologico Monzino-IRCCS, 20138 Milan, Italy. araucci@ccfm.it; Tel.: +39-0258002802; Fax: +39-0258002342.

Supplementary Materials

Table S1. List of antibodies used for Western Blot analysis.

Antibody	Company	Catalog N°	Concentration/Dilution
JAK1	Cell Signaling Technology	#97999	1:1000
Phospho-JAK1	Cell Signaling Technology	#97999	2 µg/ml
JAK2	Cell Signaling Technology	#97999	1:1000
Phospho-JAK2	Cell Signaling Technology	#97999	1:1000
JAK3	Cell Signaling Technology	#97999	1:1000
pJAK3	Cell Signaling Technology	#97999	1:1000
TYK2	Cell Signaling Technology	#97999	1:1000
pTYK2	Cell Signaling Technology	#97999	1:1000
Phospho-STAT1	Cell Signaling Technology	#9914	0.4 µg/ml
STAT1	ThermoFischer	#PA5-81911	0.4 µg/ml
Phospho-STAT3	Cell Signaling Technology	#9914	1:1000
STAT3	ThermoFischer	#PA1-86605	5 µg/ml
Phospho-STAT5	Cell Signaling Technology	#9914	1:1000
STAT5	Life Technologies	#133600	2.5 µg/ml
Phospho-STAT6	Cell Signaling Technology	#9914	1:1000
STAT6	ThermoFischer	#MA5-15659	1:1000
Caspase 3	Abcam	#Ab32351	1:2000

Abbreviations: JAK = Janus Kinase 1; STAT = Signal Transducer and Activator of Transcription.

Table S2. Sequence of Forward and Reverse primers of genes analyzed by RTqPCR.

GENE	Forward	Reverse
RUNX2	5'-TCTGGCCTTCCACTCTCAGT-3'	5'-GACTGGCGGGGTGTAAGTAA-3'
CXCL8 (IL-8)	5'-TGCCAAGGAGTGCTAAAG-3'	5'-CTCCACAACCCTCTGCAC-3'
IL6	5'-ACAAAAGTCCTGATCCAGTTCC-3'	5'-GACTGCAGGAACTCCTTAAAGC-3'
TNFRSF11B (OPG)	5'-CAACACAGCTCACAAGAACAG-3'	5'-GAAGGTGAGGTTAGCATGTCC-3'
CCL2 (MCP-1)	5'-CCCAAAGAAGCTGTGATCTTC-3'	5'-ATTCTTGGGTTGTGGAGTGAG-3'
UBC	5'-TCTTCGTGAAGACTCTGACTGG-3'	5'-GGAGGGATGCCTTCCTTATC-3'
ZNF527	5'-GGAACTTGGTATGGCTTGGACT-3'	5'-CTCCCAGTCTGCACAGTGAC-3'

Abbreviations: *CCL2* = C-C Motif Chemokine Ligand 2; *CXCL8* = C-X-C Motif Chemokine Ligand 8; *IL6* = Interleukin 6; *RUNX2* = Runt-Related Transcription Factor 2; *TNFRSF11B* = TNF Receptor Superfamily Member 11b; *UBC* = Ubiquitin C; *ZNF527* = Zinc Finger Protein 527.

Supplementary Figures and Legends

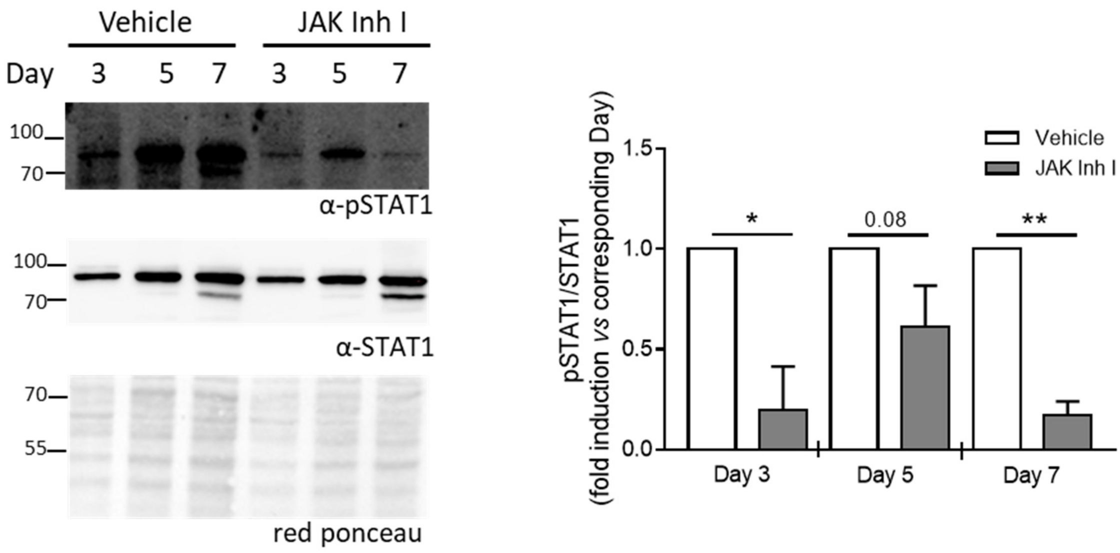

Figure S1. Inhibition of STAT1 phosphorylation by JAK inhibitor I during HASMCs calcification. HASMCs were cultured in calcification medium in presence of 0.5 μM JAK inhibitor I (JAK Inh I) or equal volume of DMSO (Vehicle). (Left panels) Representative western blot images of phosphorylated and total STAT1 of HASMCs cultured in osteogenic medium for 3, 5, and 7 days (Day). Red ponceau staining was used to normalize protein loading. (Right panels) Quantification of phosphorylated/total STAT1. Paired t test (Day 3, 5, 7 JAK Inh I *vs* corresponding Day 3, 5, 7 Vehicle); *, $p < 0.05$; **, $p < 0.01$; $n = 3$.

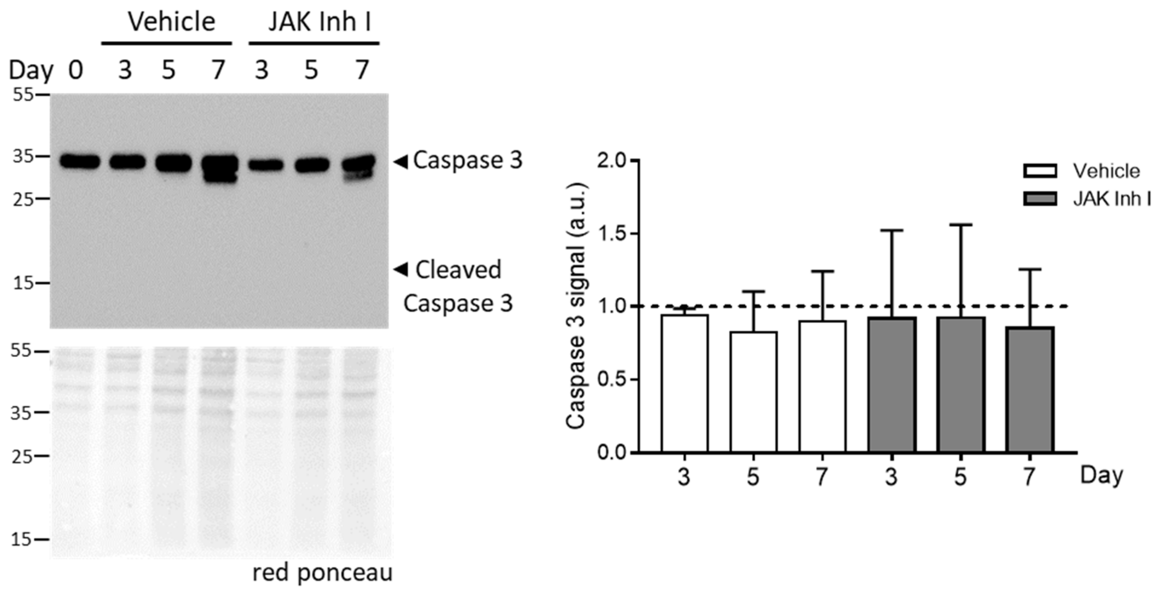

Figure S2. Apoptosis during HASMCs calcification. HASMCs were cultured in calcification medium in presence of 0.5 μM JAK inhibitor I (JAK Inh I) or equal volume of DMSO (Vehicle). (Left panels) Representative western blot images of Caspase 3 of HASMCs cultured in osteogenic medium for 0, 3, 5, and 7 days (Day). Red ponceau staining was used to normalize protein loading. (Right panels) Quantification of Caspase 3. One-way Anova with Bonferroni post hoc test (Day 3, 5, 7 Vehicle *vs* Day 0). Paired t test (Day 3, 5, 7 JAK Inh I *vs* corresponding Day 3, 5, 7 Vehicle); n = 3.

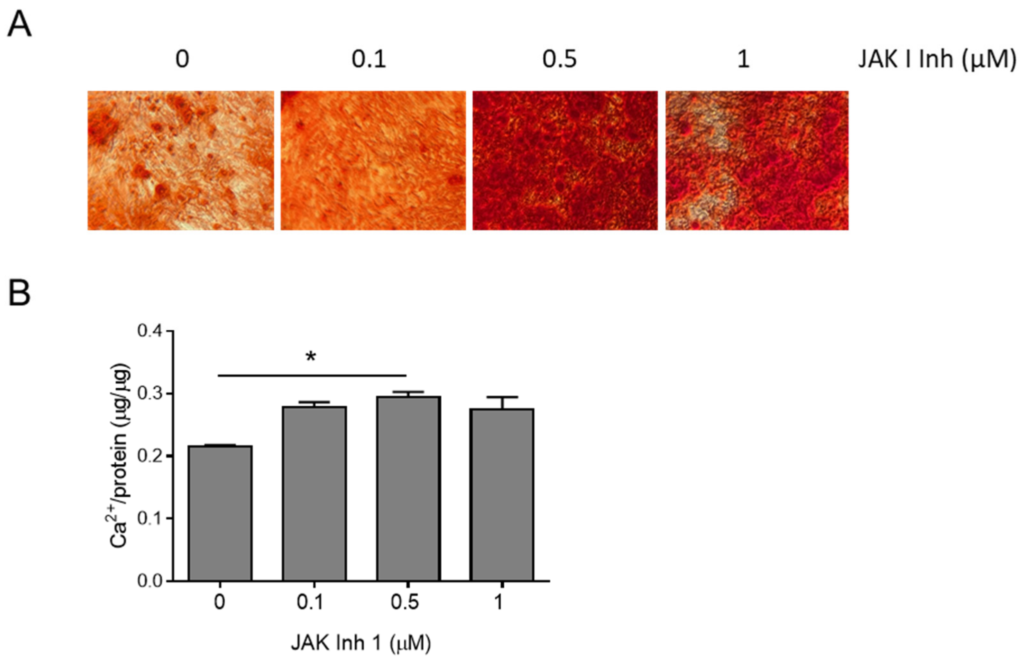

Figure S3. Dose-response effect of JAK inhibitor I on HASMCs calcification. (A-B) HASMCs were cultured in calcification medium in presence of indicated concentration of JAK inhibitor I (JAK Inh I) or DMSO (0). **(A)** Representative images of HASMCs cultured in calcification medium for 7 days and after Alizarin Red staining (red). **(B)** Calcium content quantified by colorimetric analysis and normalized on protein content. t- test; *, $p < 0.05$; $n = 2$.

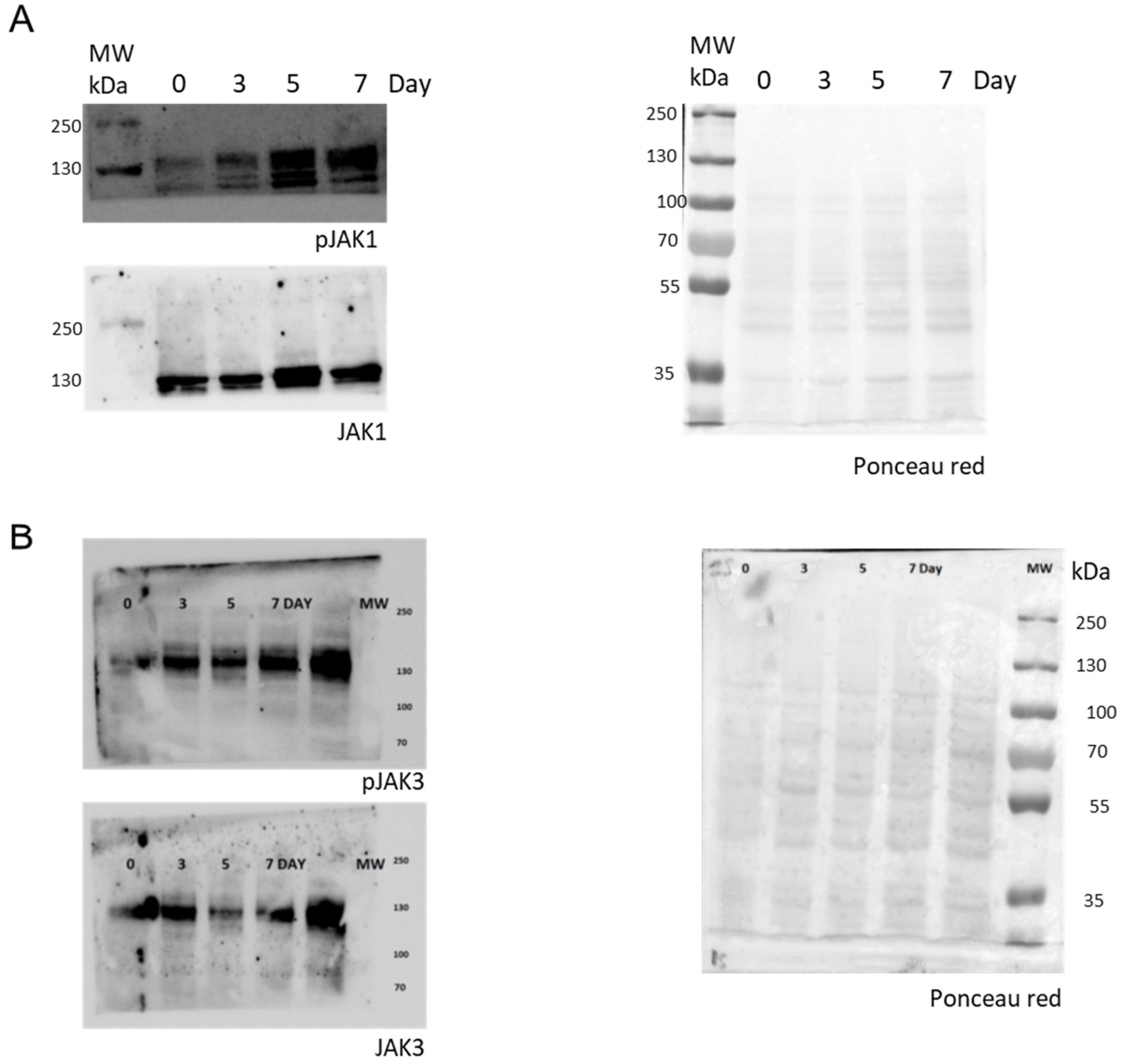

Figure S4. Original images of Figure 2. (A-B) Western blot images of phosphorylated (pJAK) and total JAK members of HASMCs cultured in osteogenic medium for 0, 3, 5, and 7 days (Day). Ponceau red staining was used to normalize protein loading. MW = Molecular weight.

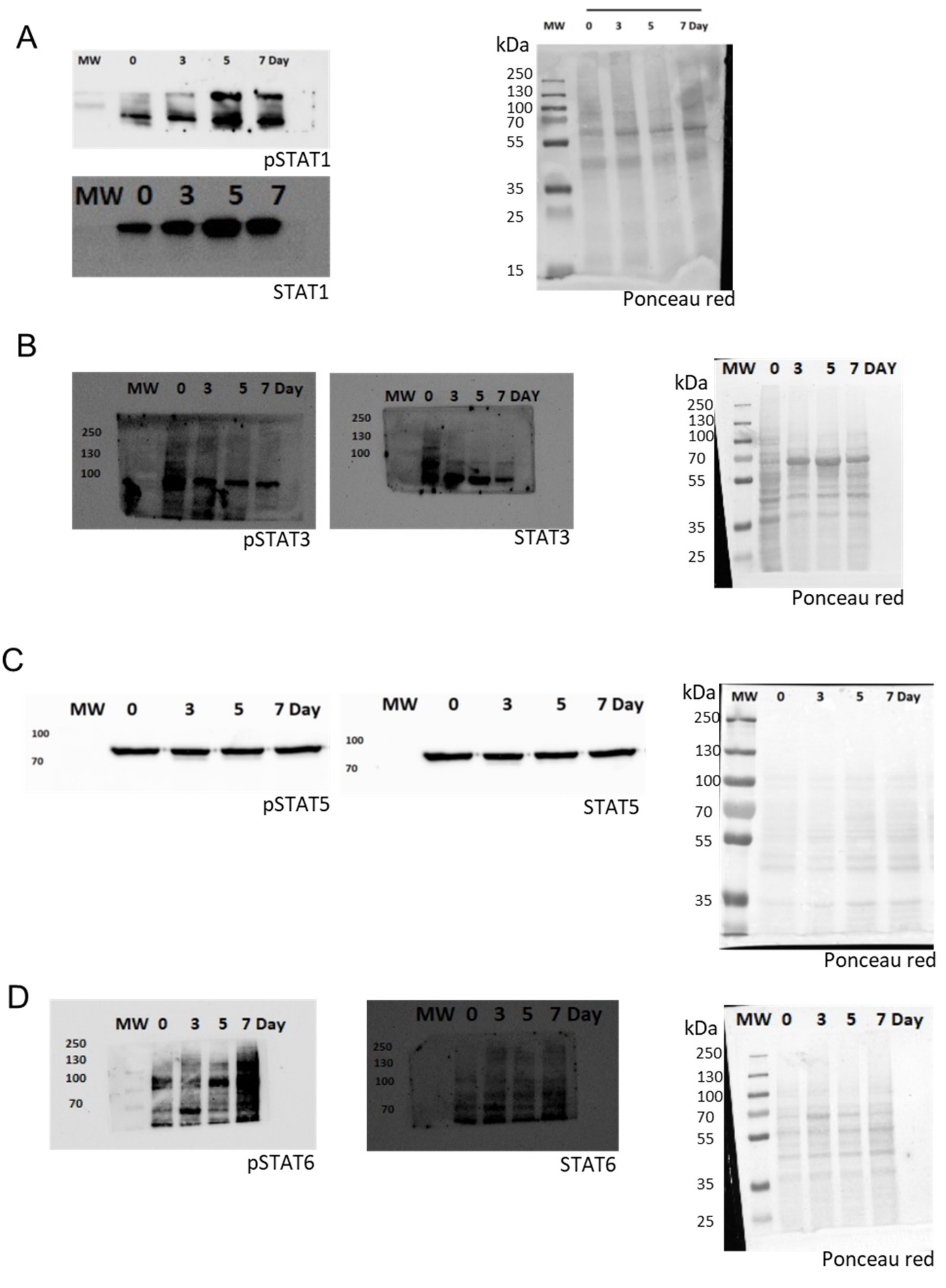

Figure S5. Original images of Figure 3. (A-D) Western blot images of phosphorylated (pSTAT) and total STAT members of HASMCs cultured in osteogenic medium for 0, 3, 5, and 7 days (Day). Ponceau red staining was used to normalize protein loading. MW = Molecular weight.
